# Promoting OPD Patient Satisfaction through Different Healthcare Determinants: A Study of Public Sector Hospitals

**DOI:** 10.3390/ijerph16193719

**Published:** 2019-10-02

**Authors:** Abid Hussain, Muhammad Asif, Arif Jameel, Jinsoo Hwang, Noman Sahito, Shahida Kanwel

**Affiliations:** 1 School of Public Affairs, Zijingang Campus, Zhejiang University, Hangzhou 310058, China; abidhusssain02@gmail.com (A.H.); asif.ma015@gmail.com (M.A.); arifjamil24@gmail.com (A.J.); 2 The College of Hospitality and Tourism Management, Sejong University, 98Gunja-Dong, Gwanjin-Gu, Seoul 143–747, Korea; 3 Department of City & Regional Planning, Mehran University of Engineering & Technology, Jamshoro 76062, Pakistan; noman_sahito@yahoo.com; 4 Tourism & Hotel Management, School of Management, Zhejiang University, Hangzhou 310058, China; shahidakanwel@yahoo.com

**Keywords:** medical equipment, information received, distance from hospital, physical infrastructure, public hospitals, Pakistan

## Abstract

Patient satisfaction is a core tool for measuring the performance of the hospitals as well as the service provider and the services that they are providing to the patients. The aim of this research is to evaluate how information received, medical equipment, distance from the hospital, and physical infrastructure influenced patient satisfaction at public hospitals in Southern Punjab, Pakistan. An exploratory research technique was used. We distributed 700 questionnaires through a random method, and 579 provided proper responses. A confirmatory factor analysis (CFA) and a regression analysis were used for the data analysis. The study results illustrated that medical equipment, information received, distance from the hospital, and physical infrastructure had significantly positive impacts (*p* = 0.001) on patient satisfaction. To promote higher level of satisfaction, there is a need to take appropriate steps for improvement.

## 1. Introduction

Measuring patients’ satisfaction (PS) in public hospitals has been rarely addressed in the context of Pakistan, even though it is a vital task. Unfortunately, the healthcare system of Pakistan recently has been facing a resources struggle due to corruption, economic and financial crisis in the past few years [1]. Therefore, it is compulsory to assess the level of PS, so that decision-makers can take initiatives and actions to increase the satisfaction level. The ultimate goal of the measurement is to enhance quality of life with PS [1,2]. The quality of healthcare delivery system in developed nations had a significant impact on developing countries because their healthcare delivery system and service quality provision are more advanced and they are achieving their desired goals positively in terms of satisfying their clients. As a result, they have received particular importance as a determinant of quality healthcare [3]. Patient satisfaction has been observed as a vital concept in the sector of services [4].

It is a psychological consequence that gratification is based on the experience of a product or service [5] which is actually known as the key source to measure performance through the feedback of personnel and institutions [6] as well as an essential tool for organizational financial measures [7]. Service delivery quality has a direct effect on PS [8].

The quality of service delivery in healthcare includes the facilities that people use during the initial stage and the entire procedure of admission, investigation, examination, treatment, discharge, and proper follow-ups in the hospitals [9,10]. The healthcare sector consider patient satisfaction as the main element of improvement and the quality of service delivery systems [4]. It is the point where condition-specific and general services needs meet [11].

The satisfaction of patients with technical expertise and outcomes is positively associated with the delivery enhancement efforts of hospitals [12]. The World Health Organization (WHO) also defines patient satisfaction as a core indicator out of nine significant indicators used to measure the quality of healthcare services delivery [13]. Therefore, PS has become an essential factor in service delivery and hospital performance [4]. 

For the improvement of good practices and quality services in the healthcare sector, it has been recognized that the perception of patients about the quality of services has to be assessed in depth. Service quality approaches and the tactics used should be marked as a priority of the administration of healthcare services [14,15]. Although it is difficult for the general administration of an organization, assessment of patient satisfaction is vital to point out any deficiencies in the service provider that want to be addressed quickly, so health planners can take pragmatic decisions for the improvement of services [16].

Our research evaluated the services of healthcare with outpatients (OPD) at public hospitals in Punjab, Pakistan, with patient satisfaction. There is a significant research gap in current healthcare literature about the assessment of patient satisfaction, which has been generally ignored in empirical studies [1,2,9,10]. Our study is very useful for the assessment of the Pakistani healthcare delivery system and it contributes significantly to the existing literature and research on healthcare and patient satisfaction. This study contributed to the existing literature in two ways. First, prior research employed used waiting time, doctor services, nurses’ services, medical cost, and communication to evaluate patient satisfaction [17,18,19]; we employed different factors including medical equipment, information received, distance from hospital and physical infrastructure. Secondly, previous studies are conducted in developed countries, while our study was carried out in an emerging nation Pakistan. 

## 2. Theory and Hypotheses

### 2.1. Medical Equipment

Medical equipment plays an essential role in healthcare services and is associated with patient satisfaction. It is particularly important to identify the life expectancy of each item, monitor its physical condition, and the safety of the items effects on satisfaction. The World Health Organization focuses on providing health facilities with quality to everyone under the Sustainable Development Goals 2030 [13]. Several scholars revealed that medical equipment is an important factor, which is connected to patient satisfaction. Furthermore, medical equipment conditions in the outpatient departments of public hospitals reveal efficiency, standards, and actual efforts for providing services [20].

**Hypothesis** **1** **(H1).** *The better the quality and the method of using medical equipment (ME), the higher the patient satisfaction*.

### 2.2. Information Received

The providers of healthcare services need to address patient’s issues, because patients/people need to be motivated to continue with medication follow up. The available studies advise that involvement, such as written information and verbal information, might improve medication adherence to patient drugs [21,22]. The information needs of patients may not be possibly met at all times because of contrary perceptions amid health service providers and patients with respect to the information that is required [23].

Therefore, patients and doctors exchange information during the consultation briefly for a better mutual understanding of each other and remove the cause of failure or misunderstanding about important health details regarding medication [23,24]. Moreover, patients’ requirements and search for information will prolong and change over time in reaction to their individual knowledge with the procedure of medication [23,25].

Earlier research indicated that sick people forget a considerable quantity of the provided instructions related to their health [26,27]. Previous research on healthcare discussed that the comprehension of information significantly related to satisfaction, and that information was linked to obedience with the physicians’ advice and prescriptions [28].

**Hypothesis** **2** **(H2).** *The more information the patients receive (IR), the higher the patient satisfaction.*

### 2.3. Distance from Hospitals

Previous studies analyzed the distance effect on patient satisfaction and service delivery. Goodman, et al. [29] said that specific facilities were linked with availability. The scholar defined the association among healthcare services, such as hospitalization rates, distance from home, and hospital environment with patient satisfaction [30]. Earlier studies indicated that people preferred to visit a closer hospital [31].

The distance is also considered an essential factor, which is connected with the duration of a patient’s journey to reach hospital in order to access healthcare services [32]. In case of an emergency, the patient needs healthcare as soon as possible for the sake of life survival [33]. As a result, distance is also a big barrier for patients to access healthcare services [34]. Several women faced savior problems during the delivery stage while pregnant due to the distance from hospitals [35]. Therefore, Escamilla, et al. [36] revealed in his study that distance positively influenced patient satisfaction.

**Hypothesis** **3** **(H3).** *The lesser the distance from the hospital (DFH), the higher the patient satisfaction.*

### 2.4. Physical Infrastructure

The feature of physical services deals with the perception of the patients regarding the hospital environment, cleanliness, etc. A number of scholars tried to find out the effect of the physical facilities on the quality of service delivery [37,38,39]. 

**Figure 1:**
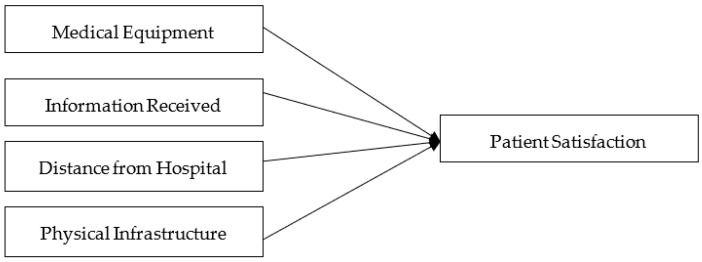
Conceptual framework of the study.

Lewis [40] described how services and physical features are very important, especially privacy, physical safety, and location. Patients focused on the interior decor, the appearance of the buildings, the layout, and the atmosphere. The items that the patients preferred the most included the appearance of the staff, the buildings, and a convenient location [41,42]. It is essential for patient health recovery that hospital administrations provide a better healing environment for the patient with skillful staff for the sake of public trust, good hospital images, and patient satisfaction [4]. All the hypothesized relations are illustrated in Figure 1.

**Hypothesis** **4** **(H4).** *The better the physical infrastructure (FI), the higher the patient satisfaction.*

## 3. Materials and Methods

### 3.1. Study Setting

This study was conducted in public hospitals of the 4 districts of southern (Bahawalpur, Bahawalnagar, Rahim Yar Khan, and Lodhran) Punjab, Pakistan. Table 1 reveals area wise population details of these districts, Table 2, illustrates area wise teaching hospitals, district hospital (DHQs), Tehsil Headquarter (THQs), Rural Health Center (RHCs) and Basic Health Unit (BHUs) [43].

### 3.2. Sample

Quantitative research was conducted on working days from May to July 2018, which were Monday to Saturday, in the outpatient department at public hospitals located in 4 southern districts (Bahawalpur, Bahawalnagar, Rahim Yar Khan, and Lodhran) in Punjab Pakistan. As per the recommendation of Saunders [45], the sample size was 700 and data were collected from patients. The 579 responses were selected for analysis, and the remaining responses were inconsistent answers and unfilled.

### 3.3. Data Collection and Instruments

The current study consists of 5 factors designed to measure respondents’ opinions on service delivery regarding the outpatient department at public hospitals. Patient satisfaction adapted from Tucker and Adams [46], was measured using 9 items, and a sample item is I have easy access to a medical specialist I need. Medical equipment (ME) comprised of 4 items with the sample item the use of up-to date medical equipment is well managed [47]. Information received (IR) included 4 items, and distance from hospital (DFH) included 3 items from Thi, et al. [48]. The IR and DFH sample items were received from useful information on how examinations and treatments would take place and distance from a patient’s home to the hospital. The physical infrastructure (PI) included 5 items, and the sample item asked if there was a pleasant atmosphere in the ward Andaleeb [49]. The patient’s demographic characteristics for the study contained education; family income, marital status, and age (see Table 3 for more details).

A 5-point Likert’s scale was used to evaluate all items (excluding the demographic details), where 1 = strongly disagree and 5 = strongly agree. For the patients convenience, such as patients who did not have any formal education, the questions were orally asked in the local language, which is Sariki, to obtain good responses [50].

## 4. Results

AMOS version 24.0 and SPSS was used for data analysis. The reliability of each individual item of the dimension was measured using a consistency analysis. Reliability refers to the instrument’s ability to provide consistent results with recurring uses [51,52]. Cronbach’s alpha coefficient has been widely used as a measure of reliability [53]. We found alpha (α) reliabilities for patient satisfaction = 0.92, physical infrastructure = 0.93, distance from hospital = 0.90, information received = 0.91, and medical equipment = 0.88 respectively. All these values are above the cutoff point of 0.70 as suggested by Qing, et al. [54]. Additionally, all the variables were statistically significant and positively correlated (see Table 4). Table 4 gives the summary of coefficients, the zero-order correlations, and the descriptive statistics. 

The convergent validity of the variables was evaluated by examining the factor loadings, average variance extracted (AVE). The composite reliabilities ensured the minimum cutoff at 0.60 [10], while the estimates for the AVE crossed the threshold of 0.50 [55] (See Table 5).

On the basses of the exploratory factor analysis (EFA) of the overall PS, we regulated model fit indices, which are shown in Table 6, and undertook confirmatory factor analysis (CFA). The value of the goodness of fit index (GFI) = 0.943, the comparative fit index (CFI) = 0.984, the Tucker–Lewis index (TLI) = 0.981, the incremental fit index (IFI) = 0.979, and the root mean square error approximation (RMSEA) = 0.031. All above values are by [4,56,57] recommended standards. Additionally, the overall absolute model fit indices, as indicated from the fit indices, support the validity of each constructs. 

### 4.1. Confirmatory Factor Analysis (CFA)

Confirmatory factor analysis (CFA) affirmed the relation between their underlying latent variables and the observed factors [58]. It is used to measure the loadings of items on a certain variable. The factor loading values while conducting the confirmatory factor analysis are defined in Table 7. The confirmatory factor analysis with statistical values all met the standard criteria for the adequacy of fit. The factor loadings values show the strength of the relationship of factors with their respective constructs; 1 is the maximum value for factor loading. The lowest value in this model is 0.721, and the highest factor loading value is 0.926. All factor loading values confirmed the assessment of construct validity, which is assessed by examining the loadings of the items and their convergent validity [59,60]. 

### 4.2. Hypothesis Testing Using a Multiple Regression Analysis

The multiple regression analysis (see Table 8) shows that there are four predictors of overall patient satisfaction in public sector hospitals. According to the results, 37.2% (Adjusted R^2^ = 0.367, F = 74.33, and *p* = 0.001) of the variance in the outcome variable (patient satisfaction) was described by the four independent variables, which included medical equipment, information received, distance from the hospital, and physical infrastructure. To test multi-collinearity, the range of tolerance values was between 0.527 and 0.720, while a value closer to zero indicates a collinearity issue, and the range of the variance inflation factor (VIF) was between 1.238 and 1.511 (a value greater than three indicates a problem with collinearity); therefore, our results showed that multi-collinearity was not an issue in the data. The values in Table 8 revealed that all four variables were significantly and positively influenced patient satisfaction (PS).

Moreover, on the bases of the results presented in Table 8, we found significant and positive effects of medical equipment (standardized *β* = 0.250, *t* = 11.905, and *p* < 0.001) which showed that one unit increased in medical equipment will add up 0.250 units in patient satisfaction, information received (standardized *β* = 0.331, *t* = 15.762, and *p* < 0.001) revealed that a one-unit increase in information received resulted in a 0.331 unit increase in patient satisfaction. Similarly, distance from hospital (standardized *β* = 0.274, *t* = 13.700, and *p* < 0.001) and physical infrastructure (standardized *β* = 0.371, *t* = 16.130, and *p* < 0.001) also has a positive impact on patient satisfaction. Here, we found physical infrastructure as the most significant and effective factor which influenced the patient satisfaction higher than the other factors. These results support our study hypotheses (H1, H2, H3, and H4).

## 5. Discussion 

Healthcare is a fundamental aspect of any society. The aim of this study was to examine the factors, such as medical equipment, information received, distance from hospital and physical infrastructure that affect patient satisfaction in the OPD services in public hospitals. The results revealed that healthcare services provided in the hospitals regarding medical equipment have an impact on patient satisfaction. A study by Szyca, Rosiek, Nowakowska and Leksowski [20] confirmed that the condition of medical equipment and how it is used affects on patient satisfaction. The patients are facing several issues regarding medical equipment, which is not properly maintained and used, in an unhygienic condition, and unclean (dusty equipment boxes and racks).

In the healthcare system, information services are considered as the key indicators of patient satisfaction and as backbone of successful healthcare system. Research has also confirmed that information received influenced patient satisfaction [61]. The results indicate that the patients are facing several problems regarding information services. Information desks are not properly placed and working properly, and the patients are not provided information about wards, pharmacies, and laboratories. Moreover, this research also includes that patients are not receiving proper information on medication procedures, treatment follow-ups, diseases, and service mechanisms. This affects the health of the patients even more [62,63].

The hospital distance and physical infrastructure are also important factors that influence patient satisfaction in the healthcare delivery system. A study revealed that distance from the hospital has an impact on patient satisfaction [32]. Accessibility plays a big role in providing quick and effectual care. In several cases, patients cannot reach hospital on the time due to a long distance. The findings of the study also indicate that hospital physical infrastructure has an impact on patients. As per the context of public hospitals, patients are facing several issues regarding physical infrastructure, such as cleaning, poor ventilation issues, lack of proper sitting places, and poor bed conditions, and bad conditions of rest rooms Hussain, Sial, Usman, Hwang, Jiang and Shafiq [4], therefore, physical infrastructure is an important factor for patient satisfaction. All these factors are important for measuring patient satisfaction.

Khattak, et al. [64] measured “Patient Satisfaction—A Comparison between Public and Private Hospitals of Peshawar” with the constructs of “Access/Availability/Convenience, Communication with the doctor, Financial Aspect, General Satisfaction, Interpersonal manner, Time spent with the doctor and Technical quality of healthcare”. Javed and Ilyas [65] assessed the influence of patients’ expectations from healthcare service quality on their satisfaction with nursing in public and private hospitals of Pakistan through the SERVQUAL approach. According to the constructs of patient satisfaction with an outpatient department addressed in our research, we include questions regarding; medical equipment, information received, distance from the hospital, and physical infrastructure.

A study concluded that patients are more satisfied with the healthcare services if the health system is responsive in terms of respect of dignity, autonomy and prompt attention, and meeting expectations. Public sector hospitals should make sure to provide these services to patients in order to meet the needs of patients and make healthcare system more effective to facilitate the patients.

## 6. Conclusions

The findings of this study revealed significant associations between outpatient department services, such as medical equipment, information received, distance from hospitals, and physical infrastructure, and PS. When the quality of services delivery is considered as a multidimensional concept, the hospital administration and all authorities must provide invaluable tips for service delivery. The research on health services is a multidimensional construct which makes clear effective aspects of hospital services in developing or enhancing patient satisfaction.

Therefore, the administration could focus their service improvement efforts on areas of facilities that have a better impression on patient satisfaction. This study indicates that medical equipment, information received, physical services, and distance from the hospital had a great impact on overall patient satisfaction. For the sake of sustainable healthcare services, the authorities concerned must revise standard operating procedure (SOPs) for better delivery, decrease the waiting time for patients, and improve surgical operations, so hospitalization and facilities can deliver services in an effective and professional way. The hospital environment; wards, sitting areas, and sanitation should be properly maintained.

In Pakistan private health care sector is somehow responsive as indicated by few studies done in local settings but public sector is severely underutilized and there is no concept of quality improvement and quality service provision in government hospitals. To improve patient satisfaction at individual, hospital and healthcare system levels is needed and includes: introduction of concept of good care among health professionals, increase in staff competence and motivation leads to increased patient trust and satisfaction.

The majority of patient satisfaction surveys support this observation and may be more appropriate to resource-less countries as this is more cost effective than developing technical facilities. Above all, incorporation of patient satisfaction research findings at the national and local policy levels will help in enhancing patient satisfaction with the healthcare system in Pakistan.

## 7. Practical Implications

There is an immediate need for exact and authenticated data of the burden of different diseases in a population, so that all necessary steps can be taken to combat them. There must be a strong referral system between general practitioners (GPs), primary/secondary care hospitals, and tertiary care centers with comprehensive referral notes. 

Improvement of the physical infrastructure of hospitals especially for the handicapped, bedridden, and elderly patients are key steps to be taken. Increasing the number of information desks, sign boards, prominent written instructions, and color coding systems are very important for those patients who are visiting the hospital for the first time. The single most important step to be taken is that patient-to-staff ratio must be enhanced according to the standards in developed countries. Lastly, incorporating different social services networks into hospital service provisions will greatly enhance the quality of care provided and the achievement of higher scales of patient satisfaction.

## 8. Study Limitations

This study has several limitations. The first is that the data was collected in the outpatient department during working time, which is from 09:00 a.m. to 02:00 p.m. Monday to Saturday. The second limitation is that the study was held in four district public hospitals from southern Punjab. The third is that the sample number was 700. We felt this might be the case in this study and hence we avoided providing any compensation for the participants. For this research, a random sampling technique was used for data collection. This study can be expanded by increasing the variables, geographical location and sample numbers to measure further levels of patient satisfaction.

## Figures and Tables

**Table 1:** Population and area.

Area	Population	Area km^2^
Punjab Province	110,012,442	205,344
Lodhran District	1,700,620	1,790
Bahawalpur District	3,668,106	24,830
Bahawalnagar District	2,981,919	8878
Rahim Yar Khan District	4,814,006	11,880

Bureau of Statistics Punjab [44].

**Table 2:** Hospitals in districts.

Sr. No.	Teaching Hospital	District Hospital (DHQ)	Tehsil Headquarter (THQs)	Rural Health Center (RHCs)	Basic Health Unit (BHUs)
1	1	0	4	10	72
2	0	1	4	10	101
3	1	0	3	19	104
4	0	1	2	04	48
	2	2	13	33	325

Bureau of Statistics Punjab [44].

**Table 3:** Demographic characteristics.

Characteristics	Number	%
Gender		
Male	256	44.2
Female	323	55.8
Age		
Less than 20	75	12.9
20–29	99	17.1
30–39	119	20.6
40–49	149	25.7
50 and above	137	23.7
Marital status		
Married	331	57.2
Single	227	39.2
Divorced	2	0.4
Widow	19	3.2
Education		
No formal education	189	32.6
Primary/elementary school	119	20.6
Secondary/high school	123	21.2
College/university	133	23.0
Postgraduate	15	2.6
Monthly income (USD)		
Less than 1,000	140	24.2
1000–1999	111	19.2
2000–2999	123	21.2
3000–3999	101	17.4
4000–4999	63	10.9
5000 or more	41	7.1

**Table 4:** Descriptive statistics and correlations.

Factor	$\bar{X}$	SD	PS	ME	IR	DH	PI
PS	3.19	0.95	-				
ME	3.21	1.04	0.221 **	-			
IR	3.63	0.81	0.317 **	0.295 **	-		
DH	3.91	1.03	0.264 **	0.243 **	0.271 **	-	
PI	3.07	0.77	0.391 **	0.318 **	0.299 **	0.328 **	-

$\bar{X}$: Mean; SD: standard deviation; PS: patient satisfaction; ME: medical equipment; IR: information received; DH: distance from hospital; PI: physical infrastructure; ** *p* < 0.01.

**Table 5:** Composite reliability, convergent and discriminant validity.

Factor	CR	AVE	PS	ME	IR	DH	PI
PS	0.785	0.697	0.778				
ME	0.810	0.831	0.309 **	0.742			
IR	0.874	0.659	0.276 **	0.278 **	0.815		
DH	0.772	0.703	0.254 **	0.310 **	0.247 **	0.705	
PI	0.912	0.798	0.399 **	0.414 **	0.291 **	0.339 **	0.840

PS: patient satisfaction; ME: medical equipment; IR: information received; DH: distance from hospital; PI: physical infrastructure; CR: composite reliability; AVE: average variance extracted; Bold values are square root of AVE showing discriminant validity; ** 0.01.

**Table 6:** Model fit statistics.

Absolute Model Fit Indices	Value
χ^2^	1578.12
Df	846
χ^2^/df	1.865
Goodness of Fit Index (GFI)	0.943
Comparative Fit Index (CFI)	0.984
Tucker Lewis Index (TLI)	0.981
Incremental Fit Index (IFI)	0.979
Root Mean Square Error of Approximation (RMSEA)	0.031

**Table 7:** Confirmatory factor analysis (CFA).

Factor	Items	CFA Loadings	α’s
Patient Satisfaction	PS1 PS2 PS3 PS4 PS5 PS6 PS7 PS8 PS9	0.778 0.792 0.869 0.885 0.854 0.721 0.877 0.867 0.854	**0.92**
Medical Equipment	ME1 ME2 ME3 ME4	0.745 0.812 0.870 0.798	**0.88**
Information Received	IR1 IR2 IR3 IR4	0.843 0.791 0.817 0.849	**0.91**
Distance from Hospital	DH1 DH2 DH3	0.900 0.896 0.812	**0.90**
Physical Infrastructure	PI1 PI2 PI3 PI4 PI5	0.926 0.913 0.915 0.896 0.904	**0.93**

**Table 8:** Multiple regression analysis.

DV: PS	Standardized Estimates				99% CI		Collinearity	
	Β	SE	T	Sig.	LLCI	ULCI	Tolerance	VIF
Intercept	-	-	17.853	0.001	0.194	0.615	-	-
ME	0.250	0.021	11.905	0.001	0.146	0.359	0.536	1.682
IR	0.331	0.021	15.762	0.001	0.198	0.476	0.527	1.235
DH	0.274	0.020	13.700	0.001	0.267	0.614	0.682	1.471
PI	0.371	0.023	16.130	0.001	0.453	0.690	0.720	1.827
Model summary	*R* = 0.541, *R*^2^ = 0.293, F = 93.75, *p* = 0.001, Durbin-Watson (DW) = 1.79							

PS: patient satisfaction; DV: dependent variable; B: beta; SE: standard error; LLCI: lower limit concordance interval; ULCI: upper limit concordance interval.
